# Outcomes of hemi- versus whole liver transplantation in patients from mainland china with high model for end-stage liver disease scores: a matched analysis

**DOI:** 10.1186/s12893-020-00965-8

**Published:** 2020-11-20

**Authors:** LingXiang Kong, Tao Lv, Li Jiang, Jian Yang, Jiayin Yang

**Affiliations:** Department of Liver Surgery, West China Hospital of Sichuan University, Chengdu, Sichuan Province China

**Keywords:** Model for End-Stage Liver Disease Scores (MELD), Liver transplantation (LT), Living donor liver transplantation (LDLT), Split liver transplantation (SLT)

## Abstract

**Background:**

Adult hemiliver transplantation (AHLT) is an important approach given the current shortage of donor livers. However, the suitability of AHLT versus adult whole liver transplantation (AWLT) for recipients with high Model for End-Stage Liver Disease (MELD) scores remains controversial.

**Methods:**

We divided patients undergoing AHLT and AWLT into subgroups according to their MELD scores (≥ 30: AHLT, n = 35; AWLT, n = 88; and < 30: AHLT, n = 323; AWLT, n = 323). Patients were matched by demographic data and perioperative conditions according to propensity scores. A cut-off value of 30 for MELD scores was determined by comparing the overall survival data of 735 cases of nontumor liver transplantation.

**Results:**

Among patients with an MELD score ≥ 30 and < 30, AHLT was found to be associated with increased warm ischemia time, operative time, hospitalization time, and intraoperative blood loss compared with AWLT (*P* < 0.05). In the MELD ≥ 30 group, although the 5-year survival rate was significantly higher for AWLT than for AHLT (*P* = 0.037), there was no significant difference between AWLT and AHLT in the MELD < 30 group (*P* = 0.832); however, we did not observe a significant increase in specific complications following AHLT among patients with a high MELD score (≥ 30). Among these patients, the incidence of complications classified as Clavien-Dindo grade III or above was significantly higher in patients undergoing AHLT than in those undergoing AWLT (25.7% vs. 11.4%, *P* = 0.047). For the MELD < 30 group, there was no significant difference in the incidence of complications classified as Clavien-Dindo grade III or above for patients undergoing AHLT or AWLT.

**Conclusion:**

In patients with an MELD score < 30, AHLT can achieve rates of mortality and overall survival comparable to AWLT. In those with an MELD score ≥ 30, the prognosis and incidence of complications classified as Clavien-Dindo III or above are significantly worse for AHLT than for AWLT; therefore, we may need to be more cautious regarding the conclusion that patients with a high MELD score can safely undergo AHLT.

## Background

Using a hemiliver as a graft is considered hemiliver transplantation (HLT). Excluding left lateral liver transplantation (LT), standard grafts for adult split-liver transplantation (SLT) and adult living donor liver transplantation (LDLT) primarily involves right or left hemiliver. Each technique has its own related issues; for example, hepatectomies require a hemiliver graft, which will cause some damage to the living donor. Therefore, to ensure the safety of the living donor, some centers prefer that the donor retains the middle hepatic vein. Although reconstruction of the middle hepatic vein has recently become possible, the fine vasculature of donated livers requires more delicate anastomosis of the vessels and bile duct, increasing the risk of postoperative complications [1–3]. SLT has faced similar difficulties; in the 20 years following the first successful procedure, many centers have reported high rates of graft mortality and complications, as well as poor long-term survival following SLT. Most scholars believe that split livers should be considered a marginal donor liver [4]. Considering the above factors, adult HLT (AHLT) must be carried out carefully to ensure the safety of the recipient.

In February 2002, the United Network for Organ Sharing produced the Model for End-Stage Liver Disease (MELD) score as a new criteria to help prioritize graft allocation for liver transplantation (LT) [5, 6]. The MELD score of patients gradually increases during the waiting period for LT; a high MELD score is often accompanied by high mortality [7] and a poorer prognosis than patients with a low MELD score. Compared with adult whole LT (AWLT), the outcomes of AHLT are less favorable; however, whether patients with high MELD scores can safely undergo AHLT remains unclear. Therefore, the present study aimed to evaluate the utility of the MELD score for the prediction of survival and complications following AHLT and AWLT in a single center over 12 years.

## Methods

### Selection and exclusion criteria of patients

The indications for LT in our study were end-stage liver diseases and liver malignancies without hepatorenal syndrome (HRS)-acute kidney injury or chronic kidney disease (including HRS2), aged 18 years or older, and patients treated in our hospital between September 2007 and October 2019. The exclusion criteria were having undergone multiorgan combined transplantation, dual-graft LT, domino LT, or retransplantation before surgery. A total of 401 AHLTs (LDLT, n = 359; SLT, n = 42), including 231 cases with malignant tumors, 19 cases with acute liver failure, and 9 cases that were ABO incompatible, and 1241 AWLTs (malignant tumors, n = 676; acute liver failure, n = 62; ABO incompatible, n = 37) were performed in this study. SLT included 10 left and 32 right liver grafts, while LDLT included 108 left and 252 right liver grafts.

### Donor evaluation and volumetric analysis

All donors received a biochemical, coagulation, virus, electrocardiogram, and routine blood examination, as well as re-examination before operation. The retention rate of indocyanine green was measured as less than 10% in 15 min, and an intraoperative biopsy was required to assess for the presence of a fatty liver (macrovesicular steatosis of less than 10%). For living donors, the liver volume and vascular anatomy were evaluated via a three-phase enhanced computed tomography (CT) angiography and three-dimensional reconstruction system, IQQA-liver (EDDA Technology, Princeton, NJ, USA). Venous reconstruction was performed if the diameter of segment V and VIII veins and inferior right hepatic vein were more than 5 mm or the volume of venous drainage liver was more than 100 cc as estimated by the IQQA-liver software. All the AHLTs required a graft-to-recipient weight ratio of more than 0.8%. In the AHLT group, an intraoperative ultrasound was used to identify the middle hepatic vein, which was always kept in the left lobe. All SLT grafts were split in situ using brain death donors.

### Liver transplantation surgery

According to standard procedure, arterial reconstructions using microvascular surgical techniques with a running 7-0 or 8-0 polypropylene suture and the proper hepatic artery, common hepatic artery, gastroduodenal artery, or celiac trunk of recipients, can potentially be used for arterial reconstructions. Intraoperative Doppler ultrasound was used to confirm a good intrahepatic arterial flow. An end-to-end right or left portal vein anastomosis was performed using 5-0 Prolene continuous sutures. Bile duct reconstruction was performed using either duct-to-duct anastomosis with continuous suture of the posterior wall and discontinuous suture of the anterior wall, or Roux-en-Y hepaticojejunostomy.

### Postoperative protocols

All patients in our center initially received standard glucocorticoids, tacrolimus (TAC), and mycophenolate mofetil triple therapy after transplantation. The methylprednisolone was intravenously given on the first day after transplantation, then gradually reduced daily until finally being discontinued after the first week. Oral prednisone was then also tailored and discontinued within the first 3 months after transplantation. If a rejection was diagnosed, the previous dose of TAC was restored with high-dose steroid pulse therapy. For patients with stable liver function following 6 months after liver transplantation, we reduced the dosage of TAC very slowly, trying to reduce the dosage of TAC as much as possible. Heparin sodium subcutaneous injections were used to prevent thrombosis after LT; its use was stopped seven days after transplantation. The maintenance target value for activated partial thromboplastin time (APTT) should be comprehensively judged according to whether the patient exhibits postoperative bleeding; however, the duration should not exceed 70 s. The hepatic artery, portal vein, and inferior vena cava blood flow were monitored via daily color Doppler ultrasound for 7 days after the operation.

### Follow-up of patients

The mean follow-up durations for the AWLT and AHLT groups were 762 and 697 days, respectively; follow-up was routinely conducted in the outpatient clinic. They were monitored until October 2019 or until their death; their medical records were retrospectively reviewed.

### Pair match selection

While comparing the AHLT and AWLT groups, we conducted a subgroup analysis based on whether the MELD was greater than or equal to 30. Statistical computing using R software was used to accurately control for the preoperative baseline between AHLT and AWLT in the MELD ≥ 30 and MELD < 30 subgroups. Matching was carried out to minimize differences between the two groups before operation; patients were matched on variables including age^Donor^, body mass index (BMI)^Donor^, age, BMI, gender, creatinine, albumin, total bilirubin, international normalized ratio, platelet count, white blood cell count, hemoglobin, tumor, presence of hepatitis B surface antigen, and Child–Pugh score. Based on the resulting propensity score, patients in the MELD < 30 subgroup were matched 1:1 without replacement using nearest-neighbor matching within a propensity score based caliper. Since there were only 35 cases of AHLT with a MELD score ≥ 30, we matched the patients 1:3 in the MELD ≥ 30 subgroups. A corresponding Jitter plot of individual cases and histogram of standardized differences for each subgroup are presented in Additional File 1: Fig. S1 (MELD < 30 subgroup) and Additional File 2: S2 (MELD ≥ 30 subgroup), respectively.

### Definitions

EAD defined as the presence of one or more of the following postoperative laboratory: bilirubin ≥ 10 mg/dL on day 7, international normalized ratio ≥ 1.6 on day 7, and alanine or aspartate aminotransferases > 2000 IU/L within the first 7 days [8]. Primary nonfunction (PNF) was defined as nonrecoverable graft function needing urgent liver replacement during the first 7 days after LT [9].

### Statistical analysis

Overall patient survival was estimated using the Kaplan–Meier method, while differences between the two groups were determined by a log-rank test. SPSS 23.0 statistical software (SPSS Inc., Chicago, IL, USA) was used to analyze the following relevant data. Categorical data were presented as number of patients (%) and compared using Pearson’s Chi-squared and Fisher’s exact tests. Continuous variables were expressed as the mean value ± standard deviation and analyzed using a t-test and repeated measures analysis of variance; *P* < 0.05 was considered statistically significant.

## Results

### Relationship between survival rate and MELD score in liver transplant recipients without hepatocellular carcinoma

**Fig. 1:**
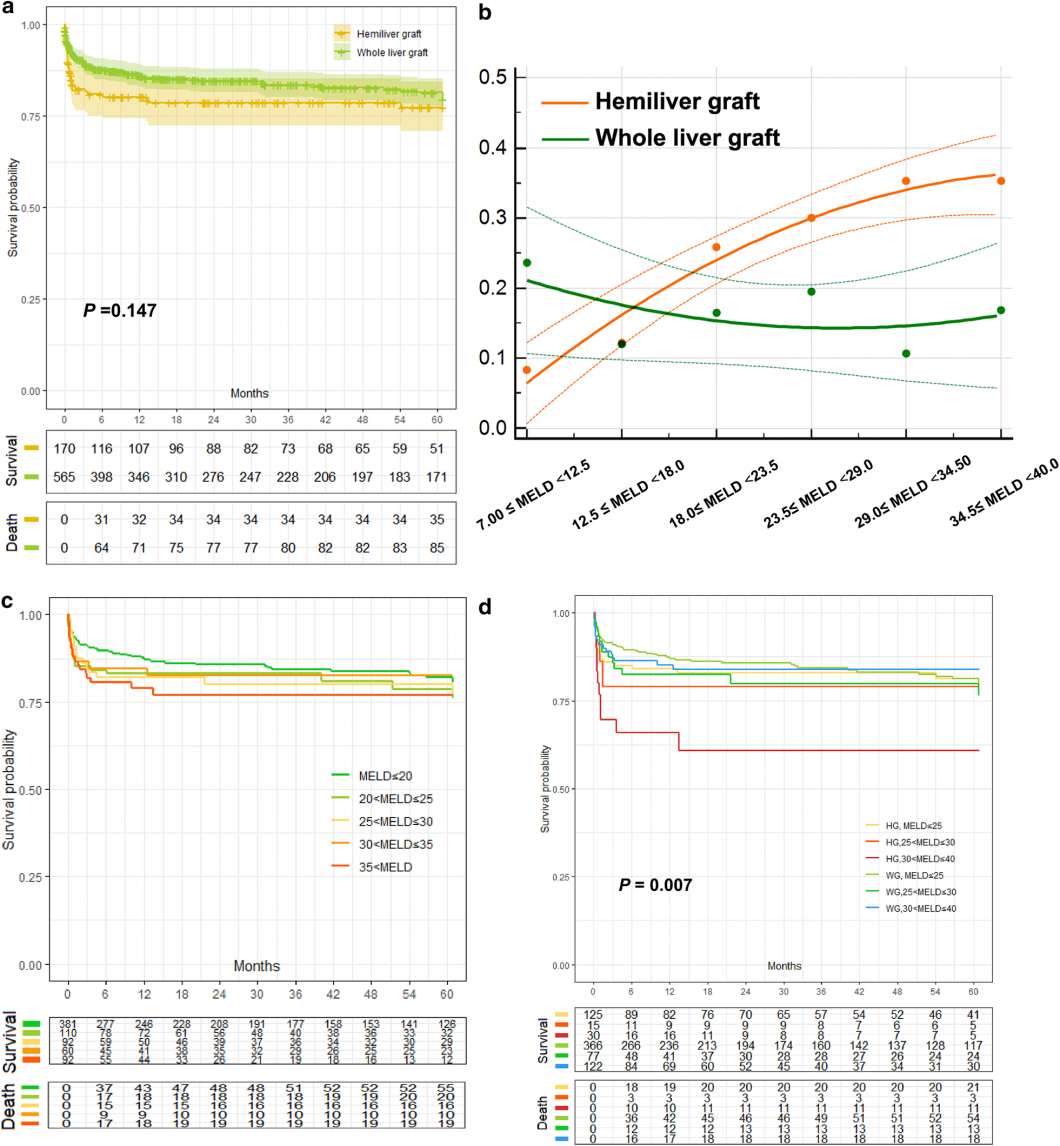
The relationship between the survival rate and MELD score in liver transplant recipients. **a** Overall survival analysis on patients who underwent AHLT (n = 170) or AWLT (n = 565). The 1-, 3-, and 5-year survival rates of AWLT were 86.2%, 83.6%, and 81.5%, respectively, while those for AHLT were 80.3%, 78.7%, and 77.4%, respectively. **b** MELD scores between 7–40 were divided into 6 segments on average as abscissa and 5-year survival rate as ordinate. The dotted line is the 95% confidence interval. **c** Overall survival rate in relation to the MELD score. **d** Analysis of the overall survival rate of half liver and whole liver groups with MELD score as subgroup. *AHLT* adult hemi-liver transplantation, *AWLT* adult whole liver transplantation, *MELD* Model for End-Stage Liver Disease

The 1-, 3-, and 5-year survival rates of AWLT without hepatocellular carcinoma (HCC) were 86.2%, 83.6%, and 81.5%, respectively, while those for AHLT without HCC were 80.3%, 78.7%, and 77.4%, respectively. The overall survival rates were not significantly different between AHLT and AWLT (*P* = 0.147). Although the overall survival rate of AHLT was found to be acceptable, a slightly partial downward shift of AHLT compared with AWLT was observed on the survival curve (Fig. 1a). Analysis of the survival rates in relation to the MELD scores are shown in Fig. 1c. Based on previous reports on the survival rate of patients below 25 points, we divided the MELD score into five groups, with ≤ 20 points as the first group and each consecutive group with an MELD score that increased by five points. The overall survival rates were significantly different for patients with an MELD score > 35 compared with those with an MELD score ≤ 20 (Fig. 1c, P = 0.067). The results of our comparison of the MELD score, when divided into six segments with the 5-year survival rate, are shown in Fig. 1b. Through fitting via R, we found that, in the MELD 23.5–29 segment, the fitting model appeared divergent; however, in the segment with a MELD score > 29, the fitting function and specific corresponding survival rate exhibited statistically significant differences (Fig. 1b). Based on the above analysis, we divided the AHLT and AWLT groups into three subgroups according to the MELD score (MELD ≤ 25, 25–30, or 30–40). The data showed that while the MELD score did not affect the AWLT, the overall survival rate decreased significantly in the case of AHLT when the MELD score was > 30 (*P* = 0.007).

### Baseline demographic and disease characteristics between the AWLT and AHLT groups

The baseline characteristics and disease features of the MELD ≥ 30 and < 30 subgroups of both the AHLT and AWLT groups are summarized in Table 1. In the post-matched model, there were no significant differences between any of the included preoperative variables between the AHLT and AWLT groups.

**Table 1:** Baseline demographic and disease features characteristics

Variables	MELD < 30			MELD ≥ 30		
	WG (n = 323)	HG (n = 323)	*P*	WG (n = 88)	HG (n = 35)	*P*
Age^Donor^ (years)	36.06 ± 11.95	36.3 ± 10.41	0.790	37.26 ± 15.75	37.66 ± 13.74	0.889
BMI ^Donor^ (kg/m^2^)	22.52 ± 2.24	22.78 ± 2.51	0.166	23.09 ± 2.33	22.73 ± 2.28	0.431
Age (years)	44.34 ± 10.15	43.76 ± 9.55	0.451	43.4 ± 10.88	44.55 ± 10.14	0.581
BMI (kg/m^2^)	22.41 ± 3.35	22.38 ± 3.1	0.923	22.76 ± 3.38	22.51 ± 2.79	0.677
Male (%)	265 (82%)	262 (81.1%)	0.761	30 (85.7%)	72 (81.8%)	0.604
CRE (μmoI/L)	72.54 ± 24.72	71.98 ± 24.33	0.774	140.78 ± 100.45	123.2 ± 100.83	0.384
ALB (g/L)	35.45 ± 6.61	35.04 ± 7.2	0.454	31.95 ± 4.77	32.32 ± 5.42	0.724
TB μmol/L	81.08 ± 125.71	73.22 ± 112.1	0.402	407.29 ± 219.85	406.91 ± 189.02	0.992
INR	1.35 ± 0.37	1.35 ± 0.37	0.960	3.11 ± 1.22	3.01 ± 1.1	0.646
PLT (10^9^/L)	103.06 ± 82.96	96.59 ± 78.73	0.310	66.97 ± 44.95	67.05 ± 43.52	0.993
WBC (10^9^/L)	5.65 ± 3.85	5.45 ± 3.41	0.486	9.3 ± 4.77	8.3 ± 4.95	0.309
HGB (g/L)	112.57 ± 27.68	112.12 ± 28.12	0.840	100.66 ± 24.02	98.77 ± 25.43	0.707
Tumor (%)	188 (58.2%)	185 (57.3%)	0.811	3 (8.6%)	8 (9.1%)	0.796
HBsAg positive (%)	231 (71.5%)	238 (73.7%)	0.537	27 (77.1%)	70 (79.5%)	0.768
Child–Pugh A (%)	68 (21.1%)	61 (18.9%)	0.491	0	0	NS
Child–Pugh B (%)	170 (52.6%)	168 (52%)	0.875	7 (20%)	10 (11.4%)	0.336
Child–Pugh C (%)	85 (26.3%)	94 (29.1%)	0.429	28 (80%)	78 (88.6%)	0.336

*BMI* body mass index, *CRE* creatinine, *ALB* albumin, *TB* total bilirubin, *INR* International Normalized Ratio, *MELD* model end-stage liver disease, *PLT* platelet, *WBC* white blood cell, *HGB* hemoglobin, *HBsAg* hepatitis B surface antigen

### Survival rate between SLT and LDLT in the post-matched model

**Fig. 2:**
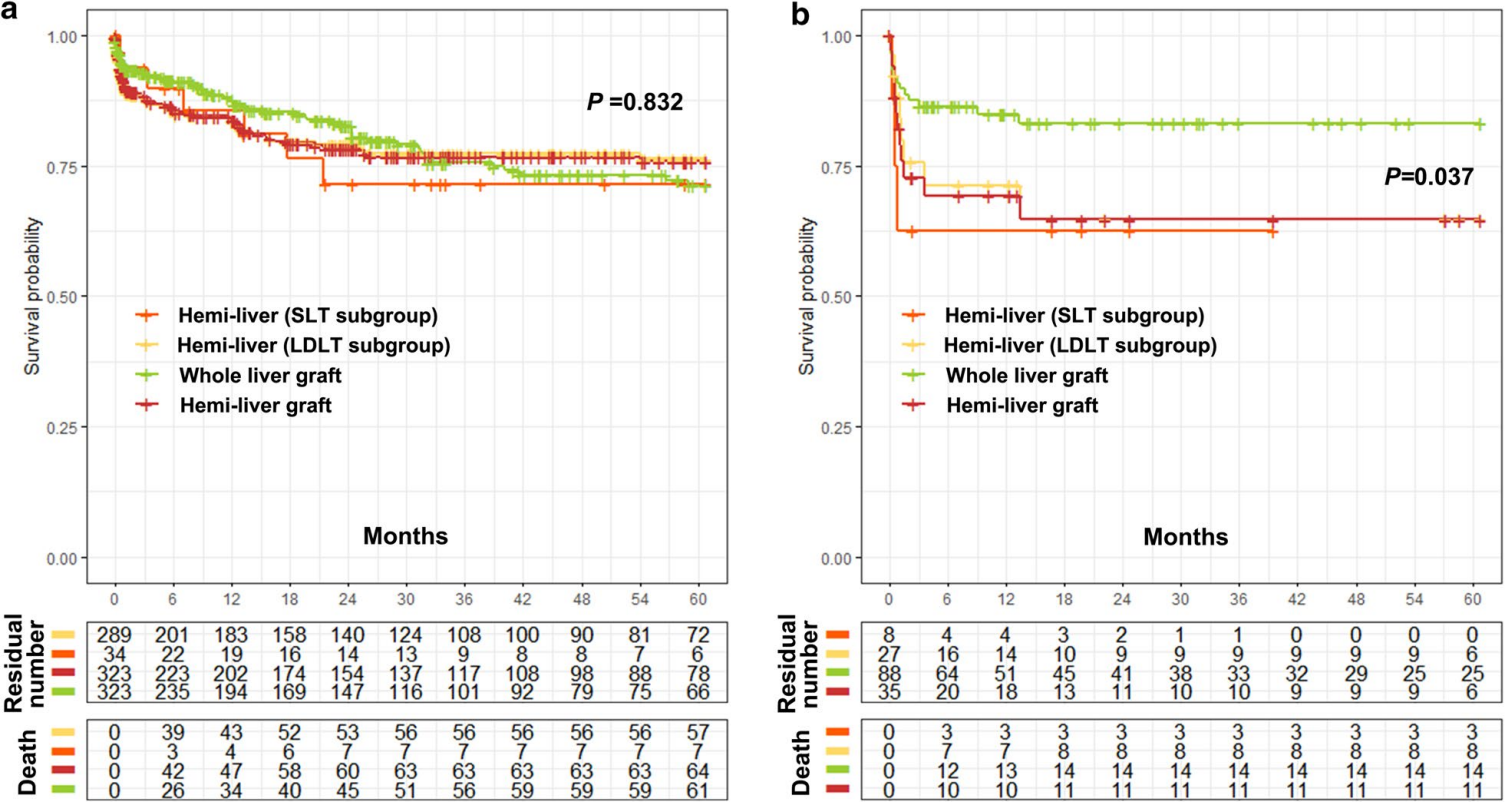
The 5-year overall survival rates of the AHLT group and the AWLT group. **a** Survival analysis of the subgroup with a MELD score < 30. **b** Survival analysis of the subgroup with a MELD score ≥ 30. *AHLT* adult hemi-liver transplantation, *AWLT* adult whole liver transplantation, *MELD* Model for End-Stage Liver Disease

The SLT and LDLT survival rates for AHLT in the different MELD subgroups are summarized in Fig. 2. In the post-matched model, the 1-, 3-, and 5-year survival rates of patients undergoing LDLT were 83.9%, 77.3%, and 76.3%, respectively, while those of patients undergoing SLT were 85.6%, 71.5%, and 71.5%, respectively. There were no significant differences in the 1-, 3-, and 5-year survival rate between the SLT and LDLT subgroups (*P* > 0.05).

### Surgical characteristics, postoperative course, and survival between the AWLT and AHLT groups

The graft to recipient weight ratio (GRWR) in the split grafts, as well as grafts from living donors, were 0.96 ± 0.10 and 0.92 ± 0.16, respectively. The intraoperative factors that showed statistically significantly differences between AHLT and AWLT groups were warm ischemia time, operative time, length of intensive care unit (ICU) stays, time of hospitalization, intraoperative blood loss, and intraoperative blood transfusion. The results of the comparison of patients undergoing AHLT and AWLT in the MELD ≥ 30 and < 30 subgroups are shown in Table 2. The reason that the warm ischemia time is significantly higher in patients undergoing AHLT than in those undergoing AWLT is that in situ perfusion technology can achieve rapid cooling when the blood supply stops; however, AHLT includes LDLT, which cannot use this technology. In the AHLT group, there are only three cases of small liver syndrome, accounting for about 0.75%. Among the HCC patients with an MELD score > 30, three (27.3%) experienced tumor recurrence within 5 years of follow-up, while for those with an MELD score < 30, there were 87 (23.3%) that experienced tumor recurrence (*P* = 0.955). There were 41 HCC recurrence cases (21.8%) in the AHLT group and 49 recurrence cases (25.0%) in the AWLT group (*P* = 0.460).

**Table 2:** Perioperative course and postoperative outcome

Variables	MELD < 30			MELD ≥ 30		
	WG (n = 323)	HG (n = 323)	*P*	WG (n = 88)	HG (n = 35)	*P*
Warm ischemia time (mins)	8.50 ± 3.82	13.02 ± 4.00	< 0.01	8.14 ± 3.29	14.57 ± 3.16	< 0.01
Cold ischemia time (hours)	3.37 ± 2.2	3.17 ± 1.21	0.137	3.39 ± 1.27	3.24 ± 3.44	0.801
Operation time (h)	8.22 ± 2.67	9.55 ± 2.01	< 0.01	8.12 ± 2.03	10.06 ± 3.2	0.002
ICU stays (days)	4.69 ± 2.51	5.24 ± 1.24	< 0.01	4.76 ± 1.55	5.17 ± 1.58	0.191
Postoperative hospital stays (days)	11.06 ± 3.78	12.38 ± 6.59	< 0.01	11.56 ± 4	15.51 ± 5.89	0.001
Intraoperative blood loss (mL)	746.16 ± 441.64	856.97 ± 351.36	< 0.01	864.77 ± 319.14	1240 ± 795.37	0.010
Blood transfusion (mL)	384.52 ± 238.28	442.11 ± 198.34	0.001	437.5 ± 205.84	717.14 ± 584.35	0.009
EAD (%)	4 (1.2%)	3 (0.9%)	NS	1 (1.1%)	1 (2.9%)	NS
PNF (%)	1 (0.3%)	0 (0.3%)	NS	0	0	NS
Postoperative infection (%)^a^	18 (5.6%)	17 (5.3%)	0.862	12 (13.6%)	6 (17.1%)	0.620
Vascular complication (%)^b^	6 (1.9%)	20 (6.2%)	0.005	3 (3.4%)	2 (5.7%)	0.938
Biliary complications (%)^c^	8 (2.5%)	14 (4.3%)	0.193	3 (3.4%)	3 (8.6%)	0.462
Intra-abdominal bleeding (%)	11 (3.4%)	22 (6.8%)	0.049	6 (6.8%)	4 (11.4%)	0.632
Clavien–Dindo ≥ Grade 3 (%)	40 (12.4%)	52 (16.1%)	0.177	10 (11.4%)	9 (25.7%)	0.047

*EAD* early allograft dysfunction, *PNF* primary nonfunction

^a^Postoperative infection including lung infection, urinary tract infection, abdominal abscess, wound infection, peritonitis, and positive blood culture

^b^Vascular complication including embolization and/or stenosis of any of the hepatic arteries, portal veins, and inferior vena cava

^c^Biliary complications including biliary stricture, biliary bleeding, or bile leakage

There were no significant differences in the incidence of early allograft dysfunction (EAD) or primary graft nonfunction (PNF) between patients undergoing AHLT and AWLT, regardless of MELD score. In the MELD ≥ 30 subgroup, although AHLT was associated with slightly higher incidences of postoperative infection, vascular complications, biliary complications, and postoperative abdominal bleeding than AWLT, this was not statistically significant. In the MELD < 30 subgroup, the incidences of vascular complications and postoperative abdominal bleeding were significantly higher in patients undergoing AHLT than in those undergoing AWLT. Specific complications and corresponding scores are summarized in Additional File 3: Table S1. Among patients undergoing AHLT with an MELD score ≥ 30, we did not observe a significant increase in the rate of any complication compared with those undergoing AWLT; however, we found that the incidence of complications with a Clavien-Dindo classification of grade III or above was significantly higher for those undergoing AHLT than for those undergoing AWLT.

In the MELD < 30 subgroup, the 5-year survival rates of AWLT and AHLT were 71.2% and 75.9%, respectively (*P* = 0.832, Fig. 2a). However, in the MELD ≥ 30 subgroup, the 5-year survival rate of patients undergoing AWLT was 64.6%, while that of patients undergoing AHLT was 83.2% (*P* = 0.037, Fig. 2b). In the MELD ≥ 30 subgroup, the survival curve showed that patient mortality peaked in the first 45 days after LT; 10–45 days post-LT was the stage that exhibited the largest difference in survival between patients undergoing AWLT and AHLT (89.8% vs. 72.7%,* P* = 0.30). Beyond 45 days, there was no significant difference in survival between AHLT and AWLT.

## Discussion

### Promising prognosis but unsatisfactory rates of use for adult hemiliver graft

In the first decade of LDLT, the 1-, 3-, and 5-year survival rates of patients undergoing LDLT without HCC were 78.8%, 73.4%, and 73.4%, respectively, while those of patients undergoing AWLT without HCC were 84.2%, 77.6%, and 70.6%, respectively [10–12]. In the case of HCC LT, although the long-term survival rate has been shown to be significantly lower than that for non-tumor patients, the long-term survival rate was not significantly different between patients undergoing LDLT or AWLT in the same era [13]. Even in the present day, these rates are supported by data at the national level and from multiple centers [14, 15]. Although SLT has been indicated to have adverse effects similar to LDLT in many reports, the ongoing improvement of surgical techniques such as in situ splitting have led to the overall survival rates of SLT and AWLT becoming similar, according to national data [16, 17]. The data from our center regarding the 1-, 3-, and 5-year survival rates revealed that AWLT has a survival similar to AHLT. In general, AHLT in adult LT patients has a good prognosis.

After the first successful adult LDLT, use of this surgery rose, peaking in 2001. However, over the past 15 years, utilization of LDLT has plateaued, while the use of SLT continues to rise slowly [18]. There is a more even distribution of WLT surgeries in centers across the world. By contrast, AHLT surgeries are more prevalent in certain transplantation centers; high-volume LDLT centers tend to be concentrated in Asia, while high-volume SLT centers are more prevalent in Europe and South Korea [18]. In mainland China, the use of AHLT exhibits the phenomenon of centralization to some transplantation centers. In our hospital, LDLT accounts for about 1/5 of the total registered number of LDLT surgeries performed in mainland China; however, the total number of LDLT surgeries performed in mainland China represents only 8% of the total number of LTs that are carried out. Although AHLT is a promising technique with a satisfactory prognosis, the worldwide application of this technique is not satisfactory [19, 20].

### MELD score and adult hemiliver transplantation

Fifteen years ago, the 1-year survival rate of patients with fulminant liver failure undergoing AHLT was reportedly only 60–70% [21]. The United Network of Organ Sharing (UNOS) recommends that an MELD score is used to accurately evaluate the basic characteristics of patients with liver disease in order to predict and assess the severity of end-stage liver disease, as well as make appropriate decisions for the allocation of donor livers for adult LT. The overall survival rate of patients with high MELD scores has been reported to be shorter than that of patients with low MELD scores [22]. The data of our present study supported this, demonstrating the 5-year survival rate of non-tumor patients with MELD scores ≤ 20 to be higher than those with MELD scores > 35.

Over the last 20 years, the outcomes of AHLT reported from many high-volume centers indicated that the long-term survival rates of patients with high or even extremely high MELD scores undergoing this procedure are not significantly different from those undergoing AWLT. In fact, some LT centers still prohibit or do not encourage AHLT for patients with a high MELD score [23, 24]; the New York State Committee on quality improvement has recommended that LDLT should not be performed for patients with MELD scores > 25. The outcomes of SLT also reflect some outcomes of LDLT, and some centers still do not perform organ splitting for patients with a high MELD score. The use of hemiliver grafts for high-MELD recipients is thus controversial [25], and there is also no universally accepted cut-off value for MELD in SLT/LDLT recipients. Early studies have reported the long-term outcomes of patients with high or very high MELD scores [10, 26–29], demonstrating that the survival rate of patients with an MELD score ≥ 36 was significantly reduced in AHLT. At present, it is generally considered safe to perform HLT in patients with MELD scores < 25; however, there is still considerable inconsistency regarding the outcomes of patients with MELD scores between 25 and 40 [30–33]. There is currently no clear definition of a high MELD score for half liver transplantation. To determine this cut-off, we analyzed the relationship between MELD score and half liver transplantation using the data from our center. Due to the possibility of tumor recurrence, we followed the previous research method and used non-HCC patients in our analysis. We found that the survival rate of patients undergoing HLT and WLT began to diverge at a MELD score of 23.5–29; with a MELD score > 29, the fitting function and specific corresponding survival rate between AHLT and AWLT was statistically significant. By comparing the survival rates of patients in each MELD score subgroup in this case–control study, we suggest that the survival results between AHLT and AWLT in patients within a MELD score of 25–30 are still acceptable; however, when the MELD score is > 30, the decrease in overall survival should lead us to carefully consider whether HLT is appropriate.

### Postoperative non-fatal complications and high MELD scores

Although it is still controversial whether AHLT is safe for patients with a high MELD score, high-volume center reports have shown that high MELD scores are relatively consistently associated with a higher incidence of complications, longer potential ICU stays, and increased hospital costs for this procedure [31, 34, 35]. Biliary complications are the most common complications of LTs, showing the biggest difference in incidence between AHLT and AWLT in the present study. In general, the incidence of biliary complications in AHLT is about twice that of AWLT [29]; although most of these patients only required medical treatment, a few may require interventional therapy. Even if surgery or interventional therapy is needed to treat biliary complications, this will not affect the survival of patients. In our study, we found that a high MELD score in patients undergoing AHLT did not cause a significant increase in biliary complications of Clavien-Dindo grade III or above. Bleeding is another common complication for which the incidence differs between HLT and WLT. The data from our center confirms that AHLT carries a higher risk than AWLT in terms of intraoperative blood transfusion and postoperative celiac hemorrhage; however, with modern medical technology and detection methods, although bleeding is common, the prognosis in most patients is usually satisfactory. By contrast, we found that the prevalence of EAD among patients with high MELD scores undergoing AHLT was 2.9% in some transplant centers. Hong et al. and Yadav et al. reported this rate to be considerably higher than in the present study (15.8% [36] and 38.3% [37], respectively); we consider this to be a result of the donor livers used in our center being too conservative. Moreover, we did not observe MELD scores to influence the incidence of EAD after operation. Even with differences in reports from various centers, the differences between EAD and PNF can be ameliorated through medical treatment. In our retrospective study, no deaths occurred due to EAD or PNF among patients with high MELD scores.

### Postoperative fatal complications and high MELD scores

Our data show that the decrease in the survival rate of patients with an MELD score ≥ 30 after AHLT usually occurs within 10–45 days; complications occurring 10–45 days after surgery are considered early LT complications, among which the major fatal complications are usually those of Clavien-Dindo grade III or above, including vascular complications, infection, renal function, and acute rejection. In our study, there were no significant differences in the incidence of acute rejection and vascular complications between AHLT and AWLT in patients with high MELD scores. In contrast with the high incidence of arterial complications reported in early studies [38], the current rate of arterial complications in adult LDLT has been reported to be low, at 1.4–4% [37, 39, 40].

Our results show that there is no significant correlation between arterial embolization and MELD score, consistent with the report of Yadav et al. [37]. Overall, the anastomotic skills of the surgeon performing HLT is no longer the main factor contributing to the incidence of complications. Interestingly, our analysis of serious complications did not reveal which specific complications were significantly increased in the case of HLT; however, the incidence of complications of Clavien-Dindo class III and above was significantly increased. We speculate that the higher death rate among patients with MELD scores ≥ 30 was a result of various factors promoting each other, eventually exacerbating the patient’s condition. Patients with a high MELD score tend to have poor liver and kidney function, while those undergoing AHLT tend to experience more bleeding and slower recovery; poor liver function will likely be accompanied by coagulation disorders, which would further increase the risk of bleeding. Massive hemorrhage and massive blood transfusion often aggravate the damage caused to renal function; once patients suffer renal failure, dialysis will cause further disorder of the internal environment, increasing the risk of infection. The high incidence of postoperative infection may, at least in part, be due to the poor preoperative conditions of patients, postoperative application of immunosuppressants, prolonged ICU stays, ventilator-assisted breathing, and disturbance of the internal environment [41–43]. However, due to the limited number of patients in the present study, this speculation could not be confirmed. Further studies with larger sample sizes are warranted to confirm this, as well as rule out any subgroup effects for patients with high MELD scores, so that patients who are not suitable for AHLT due to high MELD scores are identified and appropriate clinical decision making can be carried out.

## Conclusions

Although performing HLT is the most practical approach to address the current situation, whereby donor livers are in short supply, the rate at which this technique is adopted is unsatisfactory across the world. Here, we demonstrate that while MELD score has little effect on the outcome of AWLT, it has a significant effect on AHLT; only when the MELD score is < 30 can AHLT achieve mortality and overall survival rates comparable with AWLT. The outcome of AHLT is less favorable than AWLT in the case of a high MELD score. We were unable to identify specific complications that were significantly more frequent following AHLT; however, the incidence of complications classified as Clavien-Dindo grade III and V were significantly increased. When considering AHLT, the selection criteria that should be applied to patients with high MELD scores requires further exploration.

## Supplementary information

**Additional File 1: Fig. 1.**

**Additional File 2: Fig. 2.**

**Additional File 3: Table S1.** Classification of Postoperative Complications

## Data Availability

All related data of our center are stored in the Chinese Liver Transplant Registry, a platform for unified management of LT centers in mainland China (CLTR: http://cltr.cotr.cn). The data that support the findings of this study are available from the Chinese Liver Transplant Registry (CLTR: http://cltr.cotr.cn), but restrictions apply to the availability of these data, which were used under license for the current study, and so are not publicly available. But all related data in this study are available from the corresponding author on reasonable request.

## Abbreviations

AHLT: Adult hemiliver transplantation
AWLT: Adult whole liver transplantation
MELD: Model for end-stage liver disease
LT: Liver transplantation
LDLT: Living donor liver transplantation
SLT: Split liver transplantation
HLT: Hemiliver transplantation
HRS: Hepatorenal syndrome
CT: Computed tomography
TAC: Tacrolimus
APTT: Activated partial thromboplastin time
BMI: Body mass index
PNF: Primary nonfunction
GRWR: Graft to recipient weight ratio
ICU: Intensive Care Unit
EAD: Early allograft dysfunction
UNOS: United Network of Organ Sharing
