# Supplementary figures and images for: Outcomes of hemi- versus whole liver transplantation in patients from mainland china with high model for end-stage liver disease scores: a matched analysis
Source: BMC Surg. 2020 Nov 20;20:290. doi: 10.1186/s12893-020-00965-8 (PMC7677100; doi:10.1186/s12893-020-00965-8)

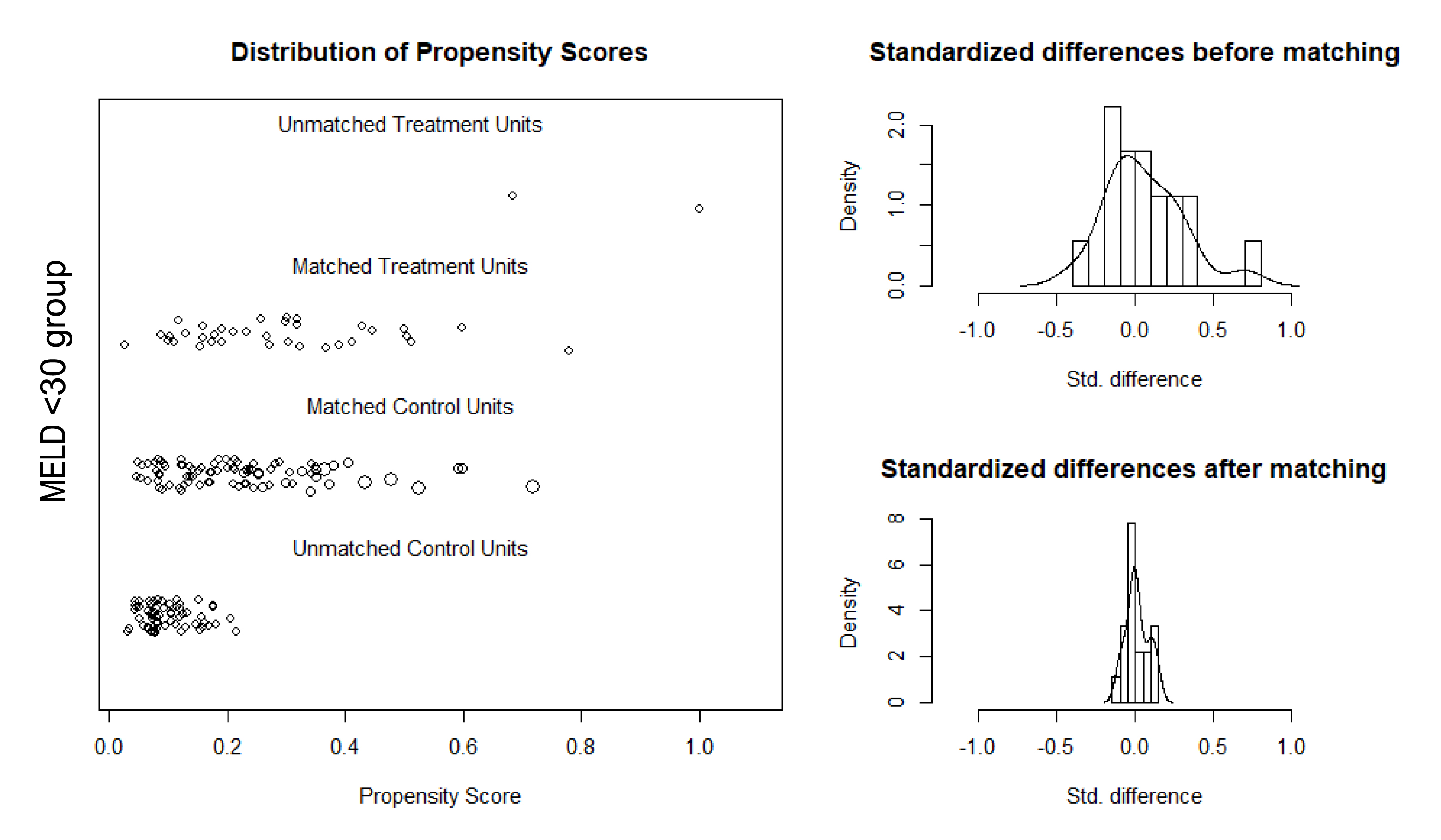

Supplement: Supplementary file 1 — Additional File 1: Fig. 1. [file 12893_2020_965_MOESM1_ESM.tif]

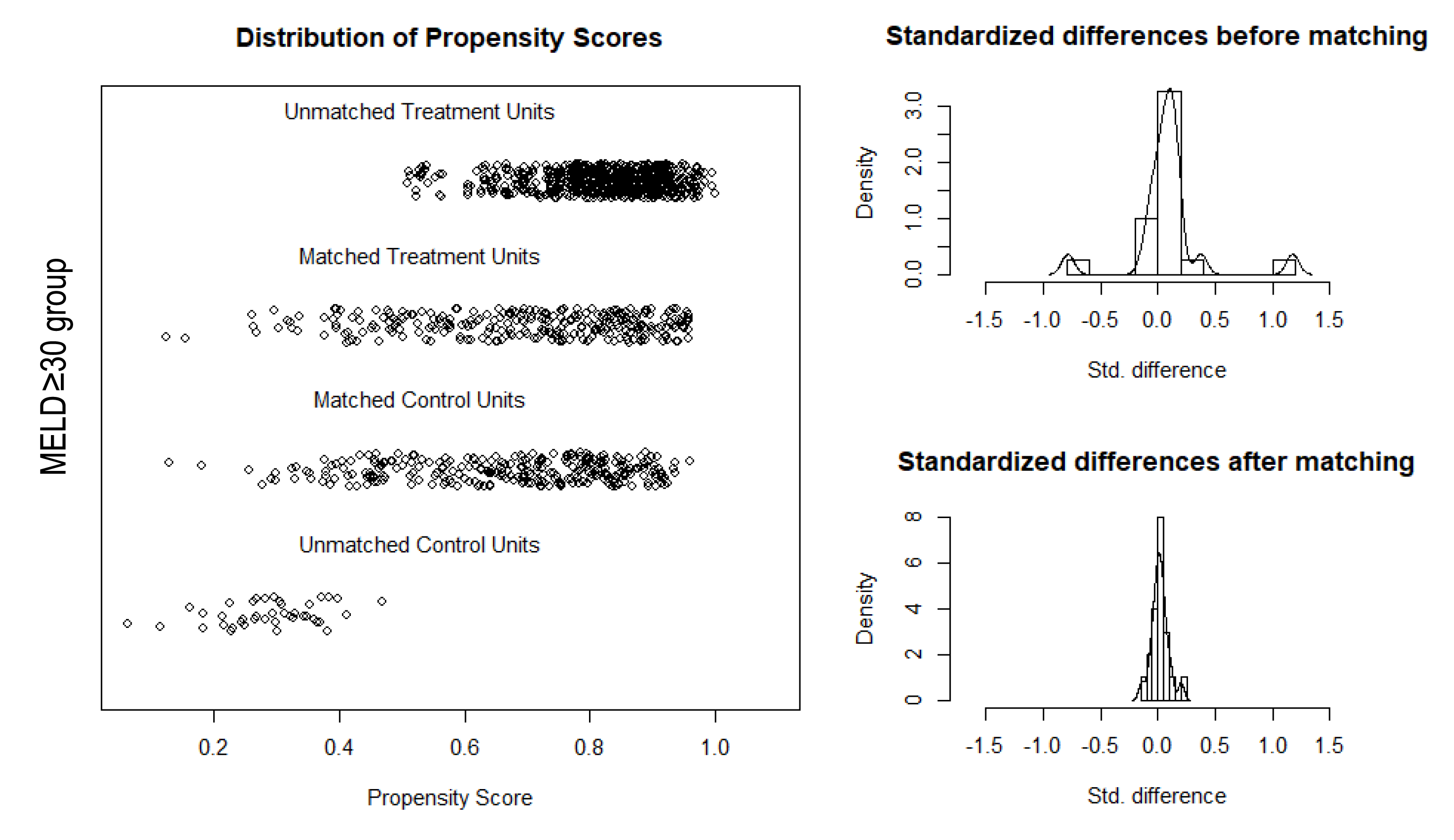

Supplement: Supplementary file 2 — Additional File 2: Fig. 2. [file 12893_2020_965_MOESM2_ESM.tif]
