# Supplementary material for: Outcomes of hemi- versus whole liver transplantation in patients from mainland china with high model for end-stage liver disease scores: a matched analysis
Source: BMC Surg. 2020 Nov 20;20:290. doi: 10.1186/s12893-020-00965-8 (PMC7677100; doi:10.1186/s12893-020-00965-8)
Supplement: Supplementary file 3 — Additional File 3: Table S1. Classification of Postoperative Complications [file 12893_2020_965_MOESM3_ESM.doc]

| Table S1. Classification of Postoperative Complications | | | | | | | |
| --- | --- | --- | --- | --- | --- | --- | --- |
| Grades | Complications* | MELD <30 | |  | MELD ≥30 | | |
|  |  | WG (n=323) | HG (n=323) | *P* | WG (n=88) | HG (n=35) | *P* |
| 3a | Pleural effusion | 5(1.5%) | 1(0.3%) | 0.219 | 1(1.1%) | 1(2.9%) | - |
| 3b | Intra-abdominal bleeding | 6(1.9%) | 13(4%) | 0.103 | 2(2.3%) | 3(8.6%) | 0.276 |
|  | Hepatic artery embolism | 3(0.9%) | 8(2.5%) | 0.128 | 0(0%) | 2(5.7%) | - |
|  | Portal/Inferior vein thrombosis / stenosis | 0(0%) | 7(2.2%) | 0.023 | 2(2.3%) | 0(0%) | - |
|  | Bile leakage/strictures | 9(2.8%) | 17(5.3%) | 0.109 | 2(2.3%) | 2(5.7%) | 0.684 |
| 4a | Single organ dysfunction (including dialysis) | 17(5.3%) | 10(3.1%) | 0.169 | 2(2.3%) | 3(8.6%) | 0.276 |
| 4b | Multiple organ dysfunction | 7(2.2%) | 7(2.2%) | 1.000 | 1(1.1%) | 1(2.9%) | - |
| 5 | Death** | 26(8%) | 28(8.7%) | 0.776 | 7(9.1%) | 6(17.1%) | 0.242 |
|  | Vascular complications^Ψ^ | 0(0%) | 0(0%) | - | 2(2.3%) | 0(0%) | - |
|  | Abdominal hemorrhage | 4(1.2%) | 5(1.5%) | 1.000 | 1(1.1%) | 2(5.7%) | - |
|  | Renal failure^Δ^ | 11(3.4%) | 8(2.5%) | 0.485 | 1(1.1%) | 1(2.9%) | - |
|  | Acute graft rejection^#^ | 6(1.9%) | 2(0.6%) | 0.286 | 3(3.4%) | 1(2.9%) | 0.684 |
|  | Infection^Φ^ | 5(1.5%) | 6(1.9%) | 0.761 | 1(1.1%) | 2(5.7%) | - |
| * Each patient may have more than one grades Ⅲ-Ⅳcomplication. ** Each patient listed only one of the most relevant causes of death. ΨVascular complications: including embolization and / or stenosis of any of the hepatic arteries, portal veins, and inferior vena cava; # Acute graft rejection: acute rejection confirmed by biopsy is a serious complication in the early stage of LT, leading to liver graft function loss; ΔRenal failure: patients need renal replacement therapy; ΦInfection including lung infection, urinary tract infection, abdominal abscess, wound infection, peritonitis, and positive blood culture. | | | | | | | |
